# Self-directed Technology-Based Therapeutic Methods for Adult Patients Receiving Mental Health Services: Systematic Review

**DOI:** 10.2196/27404

**Published:** 2021-11-26

**Authors:** Anthony Saad, Deanna Bruno, Bettina Camara, Josephine D’Agostino, Blanca Bolea-Alamanac

**Affiliations:** 1 Department of Psychiatry Women's College Hospital Toronto, ON Canada; 2 Department of Psychiatry University of Toronto Toronto, ON Canada; 3 Department of Immunology Faculty of Medicine University of Toronto Toronto, ON Canada

**Keywords:** digital therapeutics, self-directed, mental health, telehealth, technology, mobile applications, telemedicine, internet, mobile phone

## Abstract

**Background:**

Technological interventions used to treat illnesses and promote health are grouped under the umbrella term of digital therapeutics. The use of digital therapeutics is becoming increasingly common in mental health. Although many technologies are currently being implemented, research supporting their usability, efficacy, and risk requires further examination, especially for those interventions that can be used without support.

**Objective:**

This review aims to identify the evidence-based, self-directed, technology-based methods of care that can be used in adult patients after they are discharged from mental health services. The interventions reviewed are automated with no human input required (either at the patient’s or at the technology’s end), so the patients can implement them without any support.

**Methods:**

A systematic review was conducted according to the PRISMA (Preferred Reporting Items for Systematic Reviews and Meta-Analyses) and PROSPERO (International Prospective Register of Systematic Reviews) guidelines in 3 databases: PubMed, Web of Science, and OVID. The inclusion criteria were self-directed, automated, and technology-based interventions related to mental health, primarily for adults, having a solid evaluation process. The interventions had to be *self-directed*, in that the participants could use the technology without any external guidance.

**Results:**

We identified 36 papers that met the inclusion criteria: 26 randomized controlled trials, 9 nonrandomized controlled trial quantitative studies, and 1 qualitative study. The technologies used included websites, automated text messaging, phone apps, videos, computer software, and integrated voice response. There were 22 studies focused on internet-based cognitive behavioral therapies as a therapeutic paradigm compared with the waitlist, web-based human-delivered therapy, and other interventions. Among these studies, 14 used paradigms other than the internet-based cognitive behavioral therapy. Of the 8 studies comparing guided and unguided digital care, 3 showed no differences, 3 favored guided interventions, and 2 favored unguided interventions. The research also showed that dropout rates were as high as 80%, citing potential problems with the acceptability of the suggested technologies.

**Conclusions:**

There is limited research on the efficacy and suitability of self-directed technology-based care options for mental health. Digital technologies have the potential to bridge the gap between ambulatory care and independent living. However, these interventions may need to be developed collaboratively with the users to encourage their acceptability and to avoid high dropout rates.

## Introduction

### Background

Health care systems have changed dramatically over the last 50 years. The COVID-19 pandemic has specifically disrupted the traditional health care delivery model. New methods of care have been developed that can be delivered safely and that complement and improve the way treatment is provided both in and outside the physician’s office. The technological interventions used to treat illnesses and to promote health are grouped under the umbrella term of digital therapeutics [1]. There is a growing interest in digital therapeutics and their applications in the field of mental health. Digital forms of treatment have been investigated in various domains of mental health treatment, including psychotherapy, treatment of addictive behavior, medication adherence, e-therapy, obsessive-compulsive disorder, and posttraumatic stress disorder [2-6]. Maintenance of health and prevention of relapse are key concerns in mental health. For example, it is estimated that as many as one-third of patients with depression relapse during the 18 months following their recovery [7]. Mental health practitioners require not only tools that can treat their patients in the short term, but also *postdischarge* tools that will maintain health and prevent relapse. Digital therapeutics, if designed and evaluated appropriately, can be used independent of the health care providers and after having left the care of mental health services [8]. Accessibility is a key advantage of digital therapeutics. Patients who do not have access to traditional care or those who may face stigma in their communities for accessing mental health services can use digital therapeutics to obtain mental health care and avoid these problems [9]. This allows a distinctive approach to mental health practice that may improve the health of not only the individual, but also the entire population, through a better allocation of resources.

It is, therefore, essential to evaluate digital interventions regarding their usability, efficacy, and risk before they are recommended to the public [8]. Patients discharged from mental health services have access to many digital therapeutic options in the free market. They often ask physicians about these technologies and expect their technical appraisals [8]. Physicians are also understandably reluctant to endorse products that may not have been evaluated scientifically.

### Objective

Digital therapeutic methods raise issues of privacy, confidentiality, and the possible weakening of the clinician-patient relationship. Therefore, such technologies may not be accepted by potential users. It has also been suggested that the discord between the systematic nature of new technologies and the psychiatrists’ professional culture may lead to a *disruption* in mental health practice [10]. Therefore, there is a need for evidence-based research into digital therapeutics. Although other systematic reviews have examined the evidence for self-guided interventions in the past, those reviews differ in some respects to this review. Many focused on only 1 mental health condition (eg, depression), studied only 1 digital modality (eg, internet-based cognitive behavioral therapy [iCBT]), or examined interventions that were not truly independent or self-directed [11-15]. The aim of this review is to identify the self-directed digital technologies (eg, apps and websites) used to treat mental health conditions in adults with published evidence of evaluation at any level (qualitative or quantitative). The motivation was to find evidence-based *digital therapeutics* that could be used by patients after their discharge from mental health services. Once the patients are discharged, they may not remain under the guidance of mental health care professionals. Therefore, we sought the interventions that were suitable for independent use by the patients.

## Methods

The research question for this study can be summarized as follows: *What self-directed digital therapeutic options can be used by adult patients receiving psychiatric care and what is the evidence supporting their effectiveness?*

### Inclusion and Exclusion Criteria

The inclusion criteria were: (1) the studies evaluated a technology that was an internet-based or remote communication-based intervention for mental health, (2) the studies had at least 1 part or group that was self-directed (ie, the patient could perform the intervention on their own), (3) the study participants were at least 18 years of age, and (4) the studies had an evaluation component (ie, the effect, acceptability, usability, or feasibility of the technology-based intervention was studied). The studies were excluded if: (1) their primary outcome was not related to mental health or to participants with a mental health diagnosis; (2) the intervention was not completely automated (ie, required other human input for the treatment to be administered in full); (3) they had a group therapy or group forum component, as this was not deemed truly independent because group work often requires mediation and moderation by a specialist. However, the studies were not excluded if the assistance provided was carefully documented as entirely technical in nature (ie, not considered part of a therapeutic treatment).

When including the studies in this review, we enforced a strict *self-directed* criterion. Studies were only included if a digital intervention was given to at least 1 study group without any notable human support. We defined human support as any interaction between the patient and the health care team, which can be interpreted as a treatment that is psychologically beneficial. This was done to simulate the conditions of real-life practice in which the patients would use these technologies independently, without any support.

### Database Review

Three primary databases were used in this review: PubMed, Web of Science and OVID. The primary purpose of using OVID was to identify papers not captured by PubMed and Web of Science, using the *National Library of Medicine’s MEDLINE and former HealthSTAR databases* (per the OVID description page). However, as all the articles that were found in OVID either overlapped with PubMed or were ultimately excluded by our criteria, we felt assured that we had thoroughly assessed the current literature on the aforementioned topic. Appropriate keywords, including MeSH (Medical Subject Headings) terms, were used in searching the databases. The search was conducted on November 8, 2019 and included articles from the respective databases’ inception. The earliest study dated back to 1995. However, only the articles published in English were included in the study. The review broadly followed the PRISMA (Preferred Reporting Items for Systematic Reviews and Meta-Analyses) and PROSPERO (International Prospective Register of Systematic Reviews) guidelines [16]. Conventional systematic review methods were applied to this paper, including screening by title and abstract, as well as full text review. We applied a double-coding systematic review procedure, with 2 separate reviewers assessing each article. We also followed the PRISMA guidelines and completed the checklist [16]. Automated tools, beyond conventional bibliographical methods, were not used in this study. Database software was used to organize and review the studies [17].

### Levels of Evidence

This review uses the Oxford Centre for Evidence-Based Medicine—levels of evidence (LOE) [18]. Oxford Centre for Evidence-Based Medicine has set out a methodology for systematizing the process of evaluating evidence. A number and letter grading system is used, with a designation of *1a* being the highest level (for systematic reviews with homogeneity) and a rating of *5* being the lowest (expert opinion and qualitative only studies).

## Results

### Overview

**Figure 1:**
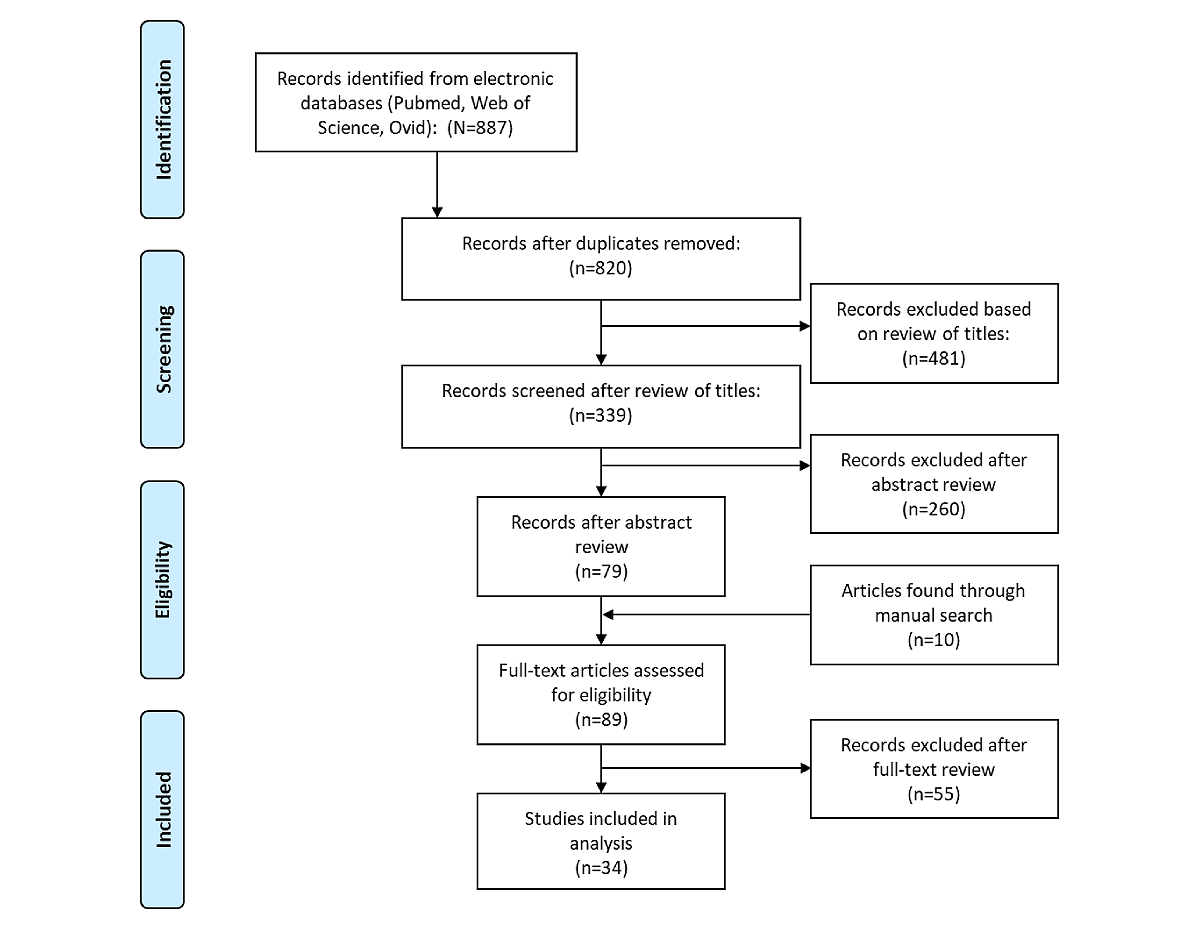
PRISMA (Preferred Reporting Items for Systematic Reviews and Meta-Analyses) flow diagram for each step of the screening process.

A total of 889 articles were identified on searching the databases. Using the PRISMA screening process, 36 studies were included in this review: 26 (72%) were randomized controlled trials (RCTs), 9 (25%) were non-RCT quantitative studies, and 1 (3%) was a qualitative study. This process flow has been illustrated in Figure 1.

Many studies were identified as iCBTs; therefore, these studies were examined as a group. Tables 1 and 2 describe the interventions studied, whereas further information about the studies has been summarized in Multimedia Appendix 1 [2,19-39] and Multimedia Appendix 2 [40-53].

**Table 1:** Description of interventions found in the 22 internet-based cognitive behavioral therapy (iCBT) studies examined.

Author (year)	Type of study	Intervention	Number and type of sessions	Description of study design
Batterham et al [19] (2018)	RCT^a^	FindMindKit: email delivered, CBT^b^- and web-based modules	18 modules in total, with 2 symptom-specific modules: for symptoms of fear disorders, distress or mood disorders, suicidal ideation, and substance disorders. Modules have scenarios and a fictional character serving as a role model and as an expert narrator. Modules were followed by a worksheet for practice.	Participants were divided into 3 groups: one receiving the personalized FindMindKit modules, one receiving the generalized modules, and an attention control group that received access to a control mental health program.
Berger et al [20] (2017)	RCT	Velibra: CBT-based web program for anxiety	6 sessions: transdiagnostic measures of treating anxiety, a form of treatment that applies similar principles across mental disorders without tailoring to specific diagnoses (eg, same treatments for GAD^c^ and social phobia). These could be tailored automatically following the user’s responses.	The intervention group had access to Velibra; the control group received access after the study was completed.
Berger et al [21] (2011)	RCT	Deprexis: self-help iCBT website	10 modules and a summary session: the content is mainly text-based, with illustrations, exercises, and user response feedback. Subsequent content is automatically tailored by the program.	All the participants had access to Deprexis. The unguided group received access without support. The guided group also received a scheduled weekly email feedback with a therapist and the freedom to contact that therapist at will.
Botella et al [22] (2016)	RCT	Smiling is fun: web-delivered, CBT-based self-help program for the treatment of depression [38].	No sessions; the website contains general multimedia, images, and an interactive platform.	Both the intervention groups had access to Smiling is fun. One of the intervention groups also had access to EEG,^d^ EKG,^e^ and ACT^f^ sensors to monitor the users’ cognitive, physiological, and physical states, as well as provide feedback. The control group did not have access to iCBT or the sensors.
Brettschneider et al [23] (2015)	Cross-sectional	Web-based text and video program for social anxiety	8 sessions: has scenarios and a fictional character serving as a role model, who also provides automatically generated feedback	All participants used the program; there was no control condition.
Christensen et al [24] (2014)	RCT	Website with CBT-based program for anxiety	10 sessions, having 1 session per week: CBT education and CBT techniques (weeks 1-7), relaxation (weeks 8-9), and physical activity promotion (week 10)	The study had 4 parts. The active condition only used the website. The control condition was a website that provided only general information on anxiety and general health. The *call* condition had a weekly telephone call, with a progress check and a reminder to use the program. The email condition had a weekly reminder via email, with similar content as the call condition.
Donker et al [25] (2013)	Randomized controlled noninferiority trial	MoodGYM: focus on dysfunctional thinking and self-esteem training [26]; CBT e-couch: deals with negative thoughts and behavioral activation; IPT^g^ e-couch: focusing on roles and interpersonal deficits	Each group used 1 of the 3 programs for a 4-week period.	This was a 3-part study that compared 2 new iCBT programs to MoodGYM (as a control) for 4 weeks.
Ebert et al [54] (2015)	RCT	Internet-based recovery training, focusing on psyhoeducation and mindfulness for the treatment of insomnia, with automated, adaptive, and tailored feedback based on user response	6 sessions	The intervention group used the program; the control group was a waitlist condition.
Gilbody et al [26] (2017)	RCT	MoodGYM: iCBT focused on dysfunctional thinking and self-esteem training	6 modules released sequentially, lasting approximately 30-45 minutes each. The participants were asked to complete 1 session per week.	The intervention group also received 8 phone calls from a graduate-level support worker, which consisted of introducing the participant to MoodGYM (first call), provide motivation and help identify the barriers to engagement (second to seventh call), and then consolidate the information and discuss the next steps (eighth call). Control group received MoodGYM without phone calls (no guidance).
Gosling et al [27] (2018)	RCT	SHUTi^h^: web- and CBT-based treatment for insomnia with modules and a sleep diary; HealthWatch: interactive lifestyle website having general health information (eg, nutrition)	N/A^i^	Two-part study comparing *SHUTi* with HealthWatch.
Hagatun et al [28] (2018)	RCT	SHUTi: see Gosling et al [27]; education website: emulates the information presented by general practitioners on insomnia	N/A	Two-part study comparing SHUTi with an education website (control group).
Hagatun et al [29] (2019)	RCT	SHUTi: see Gosling et al [27]; website: information on sleep hygiene and insomnia education	N/A	Two-part study comparing SHUTi with an informational website (control group).
Lien et al [30] (2019)	Posthoc analysis of RCT	SHUTi: see Gosling et al [27]; website: information on sleep hygiene and insomnia education	N/A	Posthoc analysis of Hagatun (2019) comparing morning versus evening persons (ie, persons with either diurnal or nocturnal sleeping habits) in the same treatment groups as the study of comparison.
Lintvedt et al [31] (2013)	RCT	MoodGYM: see [26]; BluePages: website with over 400 pages of evidence-based information on depression	N/A	Two-part study: the intervention group had access to both MoodGYM and BluePages. The control group was a waitlist condition with no intervention.
Lokman et al [32] (2017)	RCT	CDMIs,^j^ based on CBT techniques	3-4 web-based, unguided self-help modules	Two-part study comparing CDMIs to a waitlist control condition.
Loughnan et al [33] (2019)	RCT	MUMentum: pregnancy-focused, CBT-based program for antenatal depression and anxiety (illustrated, story-based exercises)	4-week unguided programs	Two-part study comparing a TAU^k^ control group with an intervention group that was provided access to MUMentum.
Mewton et al [34] (2013)	Cross-sectional	This Way Up: fully automated, unassisted web-based CBT program	N/A	All the participants were offered access to This Way Up.
Moloney et al [35] (2019)	Cross-sectional	SHUTi [27]	9 weeks	All the participants had access to SHUTi.
Noguchi et al [36] (2017)	RCT	Simplified iCBT: 5-minute exercise; sEFM^l^: based on taking time to feel negative thoughts and emotions without judgment	N/A	Three-part study comparing iCBT, sEFM, and a waiting list control group.
Proudfoot et al [37] (2013)	RCT	myCompass: fully automated self-help monitoring system, completed via mobile phone or computer	12 modules, 10 minutes each in length, comprised of skill-building activities.	Participants were randomly placed in 3 groups: the myCompass intervention group; the attention control group, which received a control mental health program; and the waitlist group that did not receive access to the intervention until after the study period.
Romero-Sanchiz et al [38] (2017)	RCT	Smiling is fun: CBT-based, self-help program for depression [22]	N/A	Participants were either TSG,^m^ or were provided with LITG,^n^ which involved emails sent from a therapist offering support with the program.
Van Kessel et al [39] (2016)	RCT	MSInvigor8: CBT-based internet program	8 sessions for each group	Two-part study: MSInvigor8-Plus received regular email support from a trained clinical psychologist, while MSInvigor8-Only did not receive any support except the iCBT program.

^a^RCT: randomized controlled trial.

^b^CBT: cognitive behavioral therapy.

^c^GAD: generalized anxiety disorder.

^d^EEG: electroencephalogram.

^e^EKG: electrocardiogram.

^f^ACT: actigraphy.

^g^IPT: interpersonal therapy.

^h^SHUTi: sleep healthy using the internet.

^i^N/A: not applicable.

^j^CDMI: complaint-directed mini-intervention.

^k^TAU: treatment as usual.

^l^sEFM: simple mindfulness exercise.

^m^TSG: totally self-guided.

^n^LITG: low-intensity therapist guidance.

**Table 2:** Description of the interventions found in the non–internet-based cognitive behavioral therapy studies.

Study designs and author (year)		Treatment paradigm	Intervention technology	Description
**RCTs^a^**				
	Aardoom et al [40] (2016)	Psychoeducation	Website	Featback was a website that offered psychoeducation and general information on eating disorders, along with monitoring and tailored feedback (automatically by the program) on progress. Examined 4 dimensions: (1) body dissatisfaction, (2) concern with body weight or shape, (3) unbalanced nutrition and dieting, and (4) binge eating and compensatory behaviors. Therapist support was by email, teleconferencing, or chat.
	Bernstein et al [41] (2016)	CBT^b^	Automated text messages (and phone)	All participants received a brochure on the benefits of quitting smoking and a phone number for a smokers’ quitline. Intervention participants also received 4 weeks of nicotine patches and gum, a referral faxed to a quitline, and enrollment in SmokefreeTXT, an automatic texting library of 128 texts. Five random messages were sent per day. The evaluation used EMA,^c^ allowing users to send feedback to the automated system about mood, craving, use, or health care contact.
	Constant et al [42] (2014)	Informational only	Automated text messages	Intervention involved automated text messages starting on the first day. 13 timed text messages were sent with reminders to take medication and to provide information on bleeding, cramping, and side effects. This was compared with SOC,^d^ which was abortion counseling (eg, information on mifepristone side effects), administration of mifepristone on site, self-administration at home (1-2 days), and follow-up clinical assessment (2-3 weeks). Intervention group received both the intervention and the SOC.
	Kannisto et al [43] (2017)	Informational only	Automated text messages	Intervention was Mobile.Net, a tailored SMS text message system designed for medication adherence and outpatient care in adult patients with psychosis. Participants received semiautomatic texts for 12 months (approximately 10/month, 2-25 text messages) based on preferences. They could decide the amount, timing, frequency, and the content of the messages.
	Kleiboer et al [44] (2015)	Problem-solving therapy	Website	Five-part study that looked at varying levels of support with an internet-based, PST^e^ for depression and anxiety called Allesondercontrole, which had 5 weekly lessons with exercises guiding on problem-solving in a structured format. Condition 1 received no support, condition 2 received support upon request and condition 3 received weekly support from a coach. Condition 4 did not receive the internet-based treatment but did receive nonspecific support via chat or email. Condition 5 was a waitlist condition with access to a website containing psychoeducation about depression and anxiety.
	Mason et al [45] (2012)	Social cognitive theory	Website	Tailored advice consisted of an advice report based on several variables (eg, sex, previous quit attempts, current health, etc). Participants reported a quit date (past or future) and received a progress report 4 weeks later, which included baseline variables, quit date reminders, slip-ups, and changes in variables. Standard reports were generated using similar algorithms but with default content and modal responses and were all identical. Advice reports could be accessed and filled out at the iQUIT website.
	Pictet et al [46] (2016)	CBM^f^	Website	Three-part study that compared 2 types of cognitive bias modification programs, as well as comparing them to a waitlist condition. Both intervention groups received access to a website that introduced photographic illustrations and audio recordings depicting everyday situations, and then the patients were instructed to imagine the situations. In the Imagery CBM group, the situations always ended positively. In the Control CBM group, the situations ended positively half the time and negatively in the other half.
	Sherman et al [47] (2012)	Psychoeducation, crisis intervention model	Videos; Phone support	4-part RCT receiving either: usual care, usual care and videos, telephone counseling, or telephone counseling and videos. Usual care involved office or inpatient visits, offering education, support group access and options for referral. Psychoeducational videos were offered in the institution or in the home, with 4 phase-specific videos on coping with breast cancer diagnoses. Telephone counseling consisted of 4 phase-specific telephone calls conducted by a nurse interventionist trained in telephone counseling approaches. These were also on coping with breast cancer.
**Non-RCT quantitative studies**				
	Ahmedani et al [48] (2015)	Motivational interviewing and CBT	Computer tablet with app	Intervention was a handheld tablet in which an animated narrator interacts with the participants by user input. Responses by the participants on the tablet would lead to varying responses by the program, allowing for branching down unique pathways and feedback tailored specifically to the user. This system combined motivational interviewing and CBT models. Intervention was delivered via a handheld computer tablet with headphones.
	Kipping et al [49] (2016)	Informational only	Website	HealthCheck was a patient portal that allowed access of patients to their health care. It included access to EMR,^g^ the ability to request medication renewals on the web, view upcoming appointments and educational materials, and access to communication with the providers.
	Piette et al [50] (2013)	Informational only (medication adherence)	IVR^h^	CarePartner program (Depression Version) was an IVR system that monitored the patients’ depression symptoms using PHQ^i^-9 and provided advice to improve medication adherence and prompt clinical follow-up. Suicidal ideation led to an alert to the clinical team, instructions to call 911 or the provider, or a suicide hotline. Faxes were sent to the providers when there was a sharp rise in PHQ-9 or medication adherence problems.
	Pratap et al [51] (2018)	Cognitive control, problem-solving therapy, informational	App	Three-part study that compared 3 different self-guided phone apps for the treatment of depression. The first group used a video-game inspired app called Project EVO, a cognitive-based program designed to modulate cognitive control abilities. The second app was an iPST^j^ program. The third was daily health tips (HTips), a program designed to provide information control to overcome depressed mood through self-care and physical activity. Each app had daily reminders. All programs were self-guided
	Stein et al [52] (2012)	Informational only (medication adherence)	Computer software	CommonGround was a computerized support system that the participants could use before a medication visit. It included an introductory video about recovery from mental illness and brief videos of patients discussing their recovery. It was followed by a customized survey of the patient’s concerns, decisional balance, and trade-off exercises.
**Qualitative study**				
	Bauer et al [53] (2018)	Informational only	App	Ginger.io was a smartphone app with a web-based dashboard with notifications to complete regular clinical surveys, occasional satisfaction surveys, and with health tips (eg, self-care activities) related to depression and anxiety 3-4 times a week. The dashboard allowed for the monitoring of patient app use. Participants used this app while continuing collaborative care treatment, which was care with a general practitioner, a care manager, and a psychiatric consultant.

^a^RCT: randomized controlled trial.

^b^CBT: cognitive behavioral therapy.

^c^EMA: ecological momentary assessment.

^d^SOC: standard of care.

^e^PST: problem-solving treatment.

^f^CBM: cognitive bias modification.

^g^EMR: electronic medical record.

^h^IVR: integrated voice response.

^i^PHQ: patient health questionnaire.

^j^iPST: internet-based problem-solving therapy.

### Studies Using iCBTs

#### Overview

We identified 22 studies that used iCBT (summarized in Table 1 and Multimedia Appendix 1). All these studies incorporated an internet-based program (either via a website or via a program downloaded from a website) that followed cognitive behavioral principles for the treatment of various psychological conditions. Although the websites and programs varied in their content, they all provided access to cognitive behavioral therapy–based modules. In most programs, the users could provide feedback.

The sample sizes varied from 39 to 2413. The targeted populations had diagnoses that varied from insomnia to anxiety and depression. Most studies were RCTs in design (18/22, 82%); 14% (3/22) were cross-sectional, and 5% (1/22) were a posthoc analysis of 1 RCT. While most of the studies were rated 1b for LOE, several were rated as 2b or 2c because they had small sample sizes, had a single part with no comparison, or because they did not report the *P* values or CIs [23,34,35].

iCBT studies could be further subcategorized based on the type of comparison that was made. Of the 18 RCT studies in this category, 6 (33%) compared iCBT against a waitlist condition [2,20,31-33,37]; 5 (28%) studies compared unguided intervention with guided controls [21,24,26,38,39]; 8 (44%) studies compared iCBT with other types of interventions [19,25,27-30,36,37]; 1 (6%) study used a sensor-based approach and compared it to unguided iCBT without a sensor [22]. The 3 non-RCT studies were cross-sectional studies that used a single group to assess the feasibility, accessibility, and preliminary effectiveness of iCBT programs [23,34,35]. The iCBT studies were categorized and reviewed in more detail based on their study design.

#### Studies Using iCBT: RCTs With a Waitlist Condition Group

A total of 6 RCT studies used a single comparison: participants with access to an iCBT program against participants who either did not have access to any intervention or those who received access to the intervention after the study was completed (ie, waitlist) [20,31-33,37,54]. These studies have been reviewed in Table 1 and Multimedia Appendix 1. They were categorized separately from the other RCTs because of concerns regarding the use of waiting lists for a comparison group, as waiting lists are not comparable with placebo interventions [55]. Berger et al [20] demonstrated significant decreases in depression, anxiety, and other mental health measures when compared with a waitlist condition, with many of the participants no longer warranting the diagnoses of anxiety disorders after 6 sessions. Ebert et al [54] showed greater improvement in insomnia measures than the waitlist control group, along with more participants achieving a symptom-free state and improving on secondary measures such as depression and sleep quality. Lokman et al [32], who compared mini-cognitive behavioral therapy–based interventions to a waitlist, found a significant decrease in depression, anxiety, and sleep-related problems and a higher well-being in the intervention group. Loughnan et al [33] found that iCBT produced moderate to large effect reductions in anxiety and psychological distress compared with a waitlist condition group. Finally, Lintvedt et al [31] demonstrated lower levels of depressive symptoms, negative thoughts, and improved depression literacy compared with a waitlist control group.

#### Studies Using iCBT: RCTs Compared With Guided Interventions

A valuable approach is to compare an unguided technological intervention to a similar intervention completed under the guidance of a trained professional. Five studies in this review used this strategy and have been summarized in Table 1 and Multimedia Appendix 1 [21,24,26,38,39]. In 2011, Berger et al [21] showed an improvement in depression symptoms when compared with a waitlist condition, with no significant difference seen whether the iCBT intervention was guided by a psychotherapist or not. Christensen et al [24] did not observe improved anxiety outcomes on generalized anxiety disorder at 6- or 12-month periods on any measures (guided or unguided, iCBT, or non-iCBT treatment), but did find higher completion rates in the 3 study arms that used phone or email guidance. Gilbody and colleagues showed an improvement in depression (by PHQ-9) in the guided group over the unguided group at 4 months but not at 12 months [26]. Romero-Sanchiz et al [38] were able to show cost-effectiveness per point improvement on Beck's Depression Inventory (BDI-II) and quality-adjusted life years in the self-directed and therapist-supported groups when compared with care as usual, although it was more pronounced in the self-directed group than in the therapist-intervention group. Van Kessel et al [39] found greater reductions in fatigue in the guided group than in the unguided group but observed no significant differences in anxiety or depression.

#### Studies Using iCBT: RCTs Compared With Other Interventions

Another effective strategy to demonstrate the utility of unguided iCBT is to compare it with other psychological interventions. These studies have been reviewed in Table 1 and Multimedia Appendix 1 [19,25,27-30,36,37]. Donker et al [25] compared a specific unguided iCBT program against other unguided iCBT programs. They found that although there were no differences between the 3 groups at baseline or follow-up, their dropout rates varied. Gosling et al [27] demonstrated that the insomnia-based iCBT program sleep healthy using the internet lead to greater improvements on measures of anxiety (at posttest and at 6-month follow-up) than a website with general health tips. In 2018, Hagatun et al [28] again showed sleep healthy using the internet’s superiority over a patient education website on measures of anxiety. They also showed improvements in the measures of insomnia [29]. A posthoc analysis of this study team’s research in 2019 demonstrated that this effect was not mediated by whether a person was a morning or an evening person (ie, persons with either diurnal or nocturnal sleeping habits) [30]. Noguchi et al [36] did not find any differences between iCBT and mindfulness-based training on depression measures.

#### Studies Using iCBT: RCT Comparing Self-guided Intervention With or Without Sensors

One study used a novel intervention strategy added to iCBT, which is reviewed in Table 1 and Multimedia Appendix 1 [22]. Botella et al [22] compared 2 intervention groups. Although both groups had access to an iCBT program for depression (*Smiling is* f*un*), one group also had access to electroencephalogram, electrocardiogram, and actigraphy sensors to monitor the physiological states and to provide feedback to the users. There was also a comparison with the waitlist control. This study found that the most effective treatment for depression was the sensor group, followed by the nonsensor intervention group [22].

#### Studies Using iCBT With a Cross-sectional Study Design

Three studies used a single-part, cross-sectional study approach, and have been summarized in Table 1 and Multimedia Appendix 1 [23,34,35]. Brettschneider et al [23] observed less social anxiety and depressive symptoms over an 8-week iCBT program, with a dropout rate of 26% (10/39). Mewton et al [34] found lower scores on the measures of psychological distress and disability after a 6-course lesson, with greater adherence in older adults (>60 years old) than in younger adults. Moloney et al [35] were able to show positive and significant improvements in US women on measures of insomnia, sleep quality, depression, and the likelihood of using medication after a 6-week intervention.

### Non-iCBT Digital Therapeutic Studies

The other 14 studies in this review, which did not use iCBT, were categorized into RCTs, non-RCT quantitative studies, and qualitative studies.

#### RCTs With Non-iCBT Interventions

We identified 8 RCTs, which have been summarized in Table 2 and Multimedia Appendix 2. The RCTs were heterogeneous in nature. They encompassed several types of interventions, including websites, automated text message systems, and videos. The websites varied in content, although many of them provided access to psychoeducation modules, with some allowing users to provide their feedback. One website allowed the patients to create tailored advice reports that were generated based on user responses to preset questions [45]. Automated text messaging services allowed the participants to receive programmed text messages in the form of reminders, education, and questions about mood, craving, or use [20,41-43]. In one study, the participants could respond to text messages, allowing for ecological momentary assessment, or the immediate reporting of participants’ behaviors in real time [41]. One study provided videos for the participants to watch at home [47].

The sample size varied from n=60 to n=1758. The targeted populations included those with mental health diagnoses, as well as healthy participants who were measured using a mental health–related outcome (ie, adjustment). Although most of the studies were rated 1b for LOE, both the Bernstein and Sherman studies were given a 2b LOE rating because they had small sample sizes and the results did not report CIs [41,47].

Of the 8 RCTs, 4 (50%) had a waitlist group. Of these 4 studies, 2 (50%) had no other comparison [42,43], whereas the other 2 (50%) used at least one other comparison group [40,47]. There were 38% (3/8) of studies that compared unguided interventions with guided interventions [40,44,47]. In addition, 38% (3/8) of studies compared a novel technological approach to usual care (ie, psychoeducational websites, brochures, or usual care) [41,45,46].

Aardoom et al [40] showed that *Featback* (a website using psychoeducation principles) was superior to a waitlist condition with regard to bulimic-related psychopathology. Bernstein et al [41] demonstrated that 47% (14/30) of the intervention group showed a 7-day smoking abstinence at 1-month compared with 10% (3/30) in the control group, but this effect was less significant at 3 months (9/30, 30% vs 4/30, 13%). Constant et al [42] reported lower anxiety using the Hospital Anxiety and Depression Scale score in women in the intervention group (ie, the group receiving automated text messaging for medical abortion self-management) and that these women were better prepared for the side effects of their medication. Kannisto et al [43] looked at recruitment and attrition, finding that one-third of those screened were eligible, but two-thirds of the eligible patients refused. Many were involved in the data retrieval stage, but very few were followed up at the postal survey stage. Participants mentioned a lack of interest, lack of mobile use adherence, or the lack of ability to use a mobile device as the main influences on their adherence. Kleiboer et al [44], who examined the effect of an internet-based problem-solving therapy, found that weekly, scheduled guidance by a trained professional had a small but significant effect on depressive symptoms compared with an internet-based problem-solving therapy–only intervention. All the groups showed improvement posttreatment. Mason and colleagues, who used a website-based advice report to quit smoking, did not find a difference in prolonged smoking abstinence between a tailored advice report group and a standardized advice report control group, regardless of the socioeconomic status and whether the participants were smoking at baseline or had recently quit [45]. Pictet et al [46] showed that positive scenarios had a considerable effect on whether a treatment (here, a website-based cognitive bias modification program) was effective. Finally, Sherman et al [47] compared 4 groups that received psychoeducational videos with varying levels of support and showed that although there were improvements in all groups in adjustment to illness, there were no significant differences among the groups in the adjustment scores.

#### Non-RCT Quantitative Studies With Non-iCBT Interventions

We identified 5 non-RCT quantitative studies, as summarized in Table 2 and Multimedia Appendix 2. Study designs included feasibility, cohort, and case-control studies. The intervention types included an application program, a website, phone apps, computer software, and integrated voice response (IVR), which is a technology that allows a computer to interact with humans through the use of voice and dual tone, multi-frequency tone input via a keypad.

The application program allowed for an interactive experience between the user and the program. Notifications and surveys were also used [48]. One study used a website design that allowed the portal access to patients receiving mental health services, looking at their use, appointment keeping, and mental health recovery measures [49]. This portal included psychoeducational materials that the patients could access, as well as information about their appointments. One study used a computer software program that the participants could download at home [52]. Another study used IVR, which allowed the participants to receive automated phone calls where they could provide feedback to the system on their depression symptoms [50].

The sample size varied from n=75 to n=3158. The populations included those with mental health diagnoses, such as depression, anxiety, or psychosis. Each study examined a different outcome. Ahmedani et al [48] used scales to evaluate the interventions (eg, patient health questionnaire; PHQ-9). They found that there was a statistically significant reduction in depression scores, along with a one-third decrease in the number of patients having moderate to mild depression scores in the study cohort. Kipping et al [49] examined the use, recovery measures, and surveys for interventions. Their study showed an increased activation of service users and caregivers, with improved recovery scores (based on mental health recovery measures domains). The users were more likely to attend scheduled appointments than the nonusers. Piette et al [50] used IVR to reach the patients and measured the call completion rates between 4 different disease groups, showing that depression had the lowest call completion rates among the 4 disease groups (314/442, 71%), and the call completion rates decreased over time with the increased severity of mental health. Pratap et al [51] compared phone apps and found that they could decrease the depressive symptoms in participants, with no significant differences between the types of apps used. Stein et al [52] focused on medication adherence and found that the users of their program did not have higher medication adherence than the nonusers. Although 3 studies were rated at a 2b LOE (individual data and cohort studies), the study by Stein et al [52] was given a 3b rating because it was a case-control design that did not control the treatment allocation.

#### Qualitative Study With Non-iCBT Intervention

We identified one qualitative study, as summarized in Table 2 and Multimedia Appendix 2. Bauer et al [53] reported on a pilot feasibility and acceptability study (N=17) of Ginger.io, a smartphone app with a web-based dashboard designed to offer support and activities related to anxiety and depression in adults diagnosed with these conditions. The primary outcome was the participants’ use of the app and their survey completion rates. As a qualitative study, it was given level 5 on the LOE.

Although all 17 participants used it at first, only 6 (35%) used it for 8 weeks. Many reported feeling satisfied with the app (11/17, 67%) and found it easy to use (13/17, 77%), but few reported concerns (2/17, 13%). Despite this, 88% (15/17) of the participant completed all the weekly symptom measures before discontinuing the use of the app.

## Discussion

### Principal Findings

This review highlights the potential of digital interventions to improve mental health, as well as the areas where new research is required. The main challenges include the heterogeneity of interventions and the low-quality comparators. Patient-related issues that were identified include high dropout rates, variable efficacy, and a lack of safety evaluations.

We identified 36 studies that examined various types of digital therapeutics. Six studies (17%) had a single group and 30 (83%) used between-group comparisons. Of the 9 studies that compared a digital treatment against a waitlist, only one did not find a beneficial effect from the use of a digital therapeutic (ie, medication adherence [52]), whereas all others showed a positive effect on primary outcomes. However, these findings must be interpreted with caution; although waitlists have often been used as control conditions when assessing psychotherapy, they are not equivalent to the placebo group in a pharmacological study and may not be a suitable comparison to show effectiveness in this context [55]. Patients know that they are not receiving an intervention, that they may not receive any alternative support, or they may be frustrated by being on a waitlist. In addition, many psychiatric disorders worsen with time if left untreated. To demonstrate that digital therapeutics are a viable alternative to other treatments, future research into these programs should focus on using groups that are comparable with the intervention, instead of using the lack of any intervention as a control group.

Eight studies compared an unguided intervention to varying levels of support from a trained professional. Of these, 5 studies found a difference between the guided and unguided groups (with 3 favoring the guided interventions), and 2 did not find any difference. The treatment effects varied across studies in terms of their quality, size, and duration. One study found effects on depressive symptoms at 4 months that were not sustained at 12 months [54]. Another study found an effect on fatigue but not on depression [39]. These findings warrant further study to ascertain the specific factors that influence the effectiveness of such interventions.

There are many potential explanations for the variable effects of treatment. Perhaps the most salient point and indeed the reason why a meta-analysis could not be done is that studies are too heterogeneous. As the tables show, they differ in their target populations (eg, external population vs clinical setting), severity of disease, nature of the interventions, length and structure of the assessments used, reminders used, the cultural and ethnic backgrounds of study participants, and the social support structures that ultimately may help explain why some interventions seemed to work better than others.

Most studies compared self-guided interventions against each other or to other treatment methods, which included educational websites and traditional treatment with a mental health team. In these studies, digital therapeutic interventions were comparable with psychoeducational websites in mental health outcomes. When interventions were compared with *standard of care*, this term was usually not well-defined, preventing any conclusions to be extended outside the context of the specific study.

Of the 36 studies examined, 22 were identified as iCBT, showing the popularity of this modality of web-based psychotherapy compared with other psychological paradigms. This therapeutic approach appears to be a preferred treatment method, with many randomized studies having large sample sizes. However, many studies have compared these interventions against other digital technologies or waitlist conditions, which may not be comparable. Digital technologies are relatively new, and this fact may limit the body of research available. While iCBT is driving most of the research available, there are also other types of psychotherapy delivered digitally that warrant further study.

### Strengths and Limitations

This review focused specifically on self-directed automated interventions that patients could implement without a therapist. Independent technology-based care options can be implemented at minimal cost by the organizations and patients and can be done at home, without having to access hospital or clinic resources. The immediate availability of these technologies has important advantages regarding the access and universality of care. Their potential accessibility is far broader than other methods of care delivery, contributing to equality in health care. They can be adapted to monitor compliance and side effects of medications, and to consolidate the gains obtained through individual psychotherapy, group psychotherapy, or psychoeducation after the discharge from health care services, thus liberating the time and resources used in follow-up and potentially preventing relapse. The study participants often reported satisfaction with technology-based care and attributed benefits to the intervention [53]. The universal accessibility of these types of interventions can help reach patients who are unable to receive traditional care because of the lack of local resources or the stigma attached to mental health, thus providing a low-barrier alternative to their care [56].

The methods used to evaluate technology-based care may differ from those for traditional RCT methods. This is partly due to the way in which technology is constantly being updated. A study by Desveaux et al [57] notes that the rigor by which we evaluate health care systems is usually applied to a static, fixed intervention, which is in direct conflict with the dynamic and ever-changing nature of technology. RCTs, by definition, require blinding, and this is often not possible in psychotherapy and digital therapeutics. RCTs with blinding are the benchmark in interventions such as medication because of the lack of contextual factors affecting their use, and the context of the intervention is vital in the development of the intervention itself [57]. This includes factors related to the interaction between the technology and the user, the environmental factors, and the access to technology. Therefore, the user must be a crucial part of not only the evaluation of the intervention, but also its design. The evaluation of new digital therapeutics requires a combination of traditional RCT methodology and novel methods of evaluation that consider the adaptive nature of sociotechnological systems of technology-based care.

Research and advice on how these novel methods of evaluation should be like can currently be found in the literature, with some going as far as having designed models such as the multiphase optimization strategy and sequential multiple assignment randomized trial evaluation system [58]. These 2 approaches apply various strategies (such as the use of screening and refining phases or time-varying adaptive interventions) that account for the changing needs of digital interventions and of their target population [58]. Other researchers have described several criteria that would help evaluate digital health interventions, including the application of a multidisciplinary approach (ie, clinical and behavioral intervention, as well as computer and engineering science), or the notion of adopting the iterative approach (ie, several cycles of development and optimization), such as the accelerated creation-to-sustainability model [59,60]. Advocates of digital interventions should also consider aspects such as safety, data security, and engagement [61].

Although the aim of this study was to evaluate self-directed interventions, several studies had a part with some human-assisted support. The effect of this therapist support suggests that human interaction may play a role in the acceptability of these programs. For example, although Aardoom et al [40] found no significant differences in the improvement of eating disorder symptoms between participants in the 3 intervention groups, qualitative data suggest that participants who received therapist support showed more satisfaction with the intervention. A similar trend in which satisfaction and engagement increased with human support was found in other studies [47,53,54].

Guided treatments can have different qualitative effects than unguided treatments, but these differences are not always detected and require further study. It may be that both unguided and guided treatments are effective but in different ways and for different groups of patients. However, a notable limitation of some of the unguided interventions is that many have *technical support*, raising the question of whether a simple call from a nontrained professional provides some therapeutic benefit. In this regard, it may be that some of the study procedures that examined *unguided* interventions were not truly unguided. Therefore, these interventions might not have as much of a therapeutic effect as the studies suggested. A similar trend in which satisfaction and engagement increased with human support was found in other studies [47,53,54].

The attrition rates varied between the studies. Previous studies have shown dropout rates of up to 80% [62]. In our review, Kannisto et al [43] found that despite having only 4.8% dropout at baseline, more than half of the intervention participants (52.45%) did not complete the final study surveys. Bauer et al [53] found a similar trend: the use was 100% of the participants in the first 4 weeks but dropped off to 35% of the participants by 8 weeks (a loss of 65% in 4 weeks). Piette et al [50] found that call completion rates were lower in depression when compared with other medical conditions such as diabetes or cancer, suggesting higher dropout rates in mental health interventions. Batterham et al [19] found that only 34% of the participants completed most intervention models.

Although many studies have shown high dropout rates, there are a couple of important points to note regarding adherence to these types of interventions. First, though the dropout rates in automated community-based interventions are likely to be high, the resources needed to reach those individuals who would otherwise not have any other form of treatment are relatively low. This suggests that there is merit in delivering self-guided, low-intensity technological interventions in this subgroup. It is also worth noting that there are many reasons why the dropout rates may be high, and although it is likely that many are negative, it is entirely possible that some of these reasons could be positive. For example, if someone feels that they have benefited from the program and stops early, or if they recover before the program has concluded, they may have dropped out because of this improvement. Given the heterogeneity of the studies, we could not identify a particular patient who would benefit more from these interventions. However, as more information becomes available and more RCTs are published, the profile of an ideal patient who responds well to digital interventions can be defined [63,64].

Perhaps the greatest limitation of this review is that technology changes at a rapid pace and despite the authors’ attempts to consider a broad range of interventions, new technology-based care methods are constantly being developed and evaluated. Some of these evaluations may not have been published yet or may even remain unpublished if the results are not positive. This is an expanding field, and it is likely that more research will be published in the future.

As digital therapeutics become more available, there is a need to establish acceptable guidelines and evidence-based approaches to determine the efficiency and suitability of technology-based treatments. This need has already been recognized. The American Psychiatric Association has established the *App Evaluation Model*, which is a set of guidelines that help health care providers evaluate the safety, benefits, and potential harms of phone apps [8]. Safety issues would include: implementing safeguards on patient data and potential data sharing, scientific review of the content, and continuous evaluation of the potential harms via a user or provider feedback system [65]. Lagan et al [66] developed a framework to translate these qualitative guidelines into objective metrics using a set of standardized questions, facilitating access to critically evaluated apps for providers as well as general audiences. The development of guidelines is crucial to not only orientate clinician advice on digital therapeutics, but also to direct research to those areas that require it while ensuring safe practices for the patients.

### Conclusions

The use of technology-based interventions in health care is increasing, but there needs to be more specific outcomes to assess their efficacy over time and the maintenance of those gains. In addition, although there are many papers that examine the use of technology-based interventions, reducing the list of research articles to those that only have *fully self-guided interventions* shows that considerably less research is addressing the issues mentioned above. To be effective, the interventions should be developed in collaboration with the users. This is evidenced by the fact that dropout rates were high in most of the studies evaluated in this review. Studies on culturally and linguistically diverse communities have found that co-design of mental health services can help recognize and account for the issues related to trust, power differential, communication, and confidentiality regarding the relationships between the researchers and the communities and users of their interventions [67]. Other research has also found the benefit of co-design in children and young people, as well as in specific mental health program designs [68-70].

Current research suggests that the effectiveness of technology-based care interventions is superior to that of waitlist controls and other interventions. However, to show their effectiveness over traditional psychiatric care, the studies should use comparison groups that are comparable with the intervention studied, thus avoiding waiting lists or other nonintervention parts.

Self-directed interventions may lead to lower costs and fewer hours spent by health care providers in supporting a treatment. These interventions will also become accessible to people lacking access to health care, such as those who live far from health care centers, those who cannot travel because of disability or family commitments, or those who cannot afford traditional care. In times of crisis or quarantine, these methods of care can become crucial instruments to deliver treatment. For many people, technology-based care methods are their first point of access to care. Thus, self-directed digital therapeutics can contribute to health care equality.

## Appendix

Multimedia Appendix 1 Summary of the characteristics of the 22 internet-based cognitive behavioral therapy studies.

Multimedia Appendix 2 Summary of data extracted from non–internet-based cognitive behavioral therapy studies.

## Abbreviations

iCBT: internet-based cognitive behavioral therapy
IVR: integrated voice response
LOE: levels of evidence
MeSH: Medical Subject Headings
PRISMA: Preferred Reporting Items for Systematic Reviews and Meta-Analyses
PROSPERO: International Prospective Register of Systematic Reviews
RCT: randomized controlled trial
