# Supplementary material for: Self-directed Technology-Based Therapeutic Methods for Adult Patients Receiving Mental Health Services: Systematic Review
Source: JMIR Ment Health. 2021 Nov 26;8(11):e27404. doi: 10.2196/27404 (PMC8665378; doi:10.2196/27404)
Supplement: Multimedia Appendix 1 [file mental_v8i11e27404_app1.pdf]

Table 3. Summary of the characteristics of the 22 iCBT studies.

**Author (Year)**	**Sample**	**Outcomes**	**Evaluation**	**Results**	**LOE**
**Batterham (2018)[19]**	N=194, Tailored Group: 66, Untailored Group: 62, Control: 66. Male: 7, Female: 58, Other: 1. No ethnicity data. Inclusion criteria: Age ≥ 18, located in Australia, elevated but not severe symptoms of mental health issues. Exclusion Criteria: reported suicide attempts/high distress state in the year prior to study.	Primary Outcome: PHQ-9, GAD-7, PADIS, SOPHS. AUDIT, DUDIT, SIDAS. Secondary Outcome: Based on symptom scales Tertiary Outcome: adherence and satisfaction	3-arm RCT Tailored group: received the FitMindKit program tailored to their symptoms. Untailored group: received the general FitMindKit program. Control Group: received a non-specific HealthWatch program.	Attention control and intervention groups saw reduction in symptom composite scores ($F = 4.3$, df = 2, 61.2, $p = 0.017$). However, there was a lack of condition change over time. It was concluded that no significant differences in symptom reduction occurred between test groups ($F = 0.6$, df = 2, 61.2, $p = 0.640$).	1b
**Berger (2017)[20]**	N = 139, Treatment Group: 70, Control Group: 69. Male: 41, Female: 98, No ethnicity data. Inclusion criteria: Age ≥ 18, Internet access, German speakers, primary diagnosis of SAD, PDA or GAD, no psychosis or BPD, no change in medication 1	Primary Outcome: DASS-21, BAI, BDI-II, BSI, SF-12 (depending on participant diagnosis) Secondary Outcome: SPS, SIAS, ACQ, BSQ, MIA, MIB, PSWQ,	2-arm RCT Treatment Group: care as usual + velibra program Control Group: care as usual only	Between-group analyses of all primary outcomes showed statistically significant decreases in disease severity for the treatment group (e.g. DASS-21: 0.47 (0.13-0.81), $p<0.01$)	1b

	month before treatment. Exclusion criteria: suicidal ideation	CSQ-8		Within-group analyses suggest statistically significant changes for the treatment group (e.g. BDI-II: 0.69 (0.21-1.18), p<0.01) but not the control group (e.g. BDI-II: -0.07 (-0.40 to 0.54)) Estimate: 28.2% of SAD, 38.3% of PDA and 44.8% of GAD no longer fulfilled diagnostic criteria post-treatment; recovery was less than 10% in control.	
**Berger (2011)[21]**	N = 76, Unguided: 25, Guided: 2, Wait-list: 26; Male: 23, Female: 53, No ethnicity data. Inclusion Criteria: Age ≥ 18, internet access, BDI-II score > 13, BDI suicide item score < 2, no other psychological treatment, constant medication	Primary Outcome: BDI-II Secondary Outcome: BSI, IIP, WHOQOL-BREF, CSQ-8	3-arm RCT Unguided: access to Deprexis program Guided: access to Deprexis program as well as scheduled email contact with a trained therapist Wait-list: no intervention	Effect size (Cohen's d) l is d = 1.14 for guided vs. Wait-list, d = 0.66 for Unguided to Wait-list, and d = -0.30 for Guided vs. Unguided on BDI-II. Study reports that comparisons showed the unguided and	1b

	prescription for 1 month prior			the guided groups were significantly more improved in comparison with Wait-list (p = 0.009). No significant difference between unguided and guided (p = 0.88)	
**Botella (2016)[22]**	N = 60, I Only: 22, I + S: 19, Control: 19; All Male, No ethnicity data.<br>Inclusion Criteria: unemployed men suffering life stressors, Age ≥ 18 but ≤ 65, internet access.<br>Exclusion criteria skin allergies to sensors, personal or first-degree relative history of depression/psychosis, BDI-II ≥ 19, epilepsy, heart conditions, daily use of recreational drugs, sleep or heart drug, CVD	Primary Outcome: MINI, BDI-II, OASIS, PANAS, PSS, Expectation and Opinion of Intervention Scales, SUS	3-arm RCT<br>I Only: Intervention<br>I + S: Intervention plus sensors<br>Control: waitlist control group	The between-group effect sizes for BDI-II were, in order, I + S (0.69 [-1.72 to 0.35]), I Only (0.49 [-1.54 to 0.56]), followed by Control (0.16 [-1.16 to 1.48]).	1b
**Brettsch. (2015)[23]**	N = 39<br>Male: 14, Female: 25, No Ethnicity Data.<br>Inclusion Criteria: Age ≥ 18, internet access, SPS > 22, SIAS > 33, suicide score < 2	Primary Outcome: SPS, SIAS, LSAS<br>Secondary Outcome: BSI, BDI-II, CSQ-8	Single-arm feasibility study	29 people completed the post-assessment (26% dropout). Completers worked through 7.5	2c

	for BDI, SAD diagnosis according to SCID, no other psychological treatment, constant dose for 1 month			out of 8 modules (SD = 1.09; range = 4-8). Non-completers used 3.1 out of 8 modules (SD = 1.4, range = 1-5). Participants showed less social anxiety on all measures (composite score: t(38) = 4.93, p < .001, d = .87), as well as depressive symptoms (F (1,56) = 5.71, p =0.01) and general psychopathology (F(2, 61) = 11.41, p < 0.001).	
**Christensen (2014)[24]**	N = 558. Active: 111, Active+Email: 113, Active+Call: 110, Control:111, Control+Call: 113 . Male: 108, Female: 450, No ethnicity data. Inclusion criteria: Age 18-30, Active email and phone #, English-speaking, internet access, GAD-7≥6. Exclusion criteria: Current psychiatric	Primary Outcome: GAD-7. Secondary outcome: MINI, PSWQ, ASI, CES-D, disability measures by Days out of Role, program preference by questionnaire	5 arm RCT: Groups:“Active” - active website intervention only;“Active/Email” -active website + email reminders;“Active/Call” - active website + phone calls;“Control”- control website only;“Control/phone” - control website + phone calls.	Call or Email groups completed majority of modules (Active/email: 5.5, Active/telephone: 7.3, Control/Telephone: 8.3) Other groups finished over one-third (3.7 for both active and control). No differences among the 5	1b

	treatment or previous diagnosis of other psychiatric illness.			groups for GAD-7 scores. Significant effects for CES-D, ASI and PSWQ (lower scores) for the "Active/Email" group at post-test compared to the "Control" group (t= −2.5, P=.015; t=−3.4, P<.001; t=−2.4, P=.017 respectively). The ASI difference remained significant at 6 months (t=−2.3, P=.021).	
**Donker (2013)[25]**	N = 1843, MoodGYM: 613, CBT: 610, IPT: 620<br>Male: 509, Female: 1334, No ethnicity data<br>Inclusion Criteria: Age ≥ 18, not being treated for depression<br>Exclusion Criteria: health professionals treating or researchers studying depression	Primary Outcome: CES-D<br>Secondary Outcome: CSQ-8, treatment preference, adherence	3-arm NoninferiorityRCT:<br>MoodGYM: Group that received MoodGYM<br>CBT: Group that received internet-delivered CBT<br>IPT: Group that received internet-delivered Interpersonal Psychotherapy	There were no differences between the groups at baseline (X^2 = 3.1, p = 0.21). CBT and IPT were shown to be non-inferior to MoodGYM, with no significant differences between the 3 groups at follow-up (IPT vs.	1b

				MoodGYM: 0.18 (CI: -0.09, 0.45), CBT vs. MoodGYM: 0.12 (CI: -0.15, 0.39). Post-test showed more reliable change for CBT than IPT and MoodGYM. Dropout rates varied.	
**Ebert (2015)[26]**	N = 128, IC: 64, CC: 64, Male: 95, Female: 33, All Caucasian Inclusion Criteria: school teachers, age ≥ 18, employed, ISI ≥ 15, CI-IS ≥ 15, internet access. Exclusion Critiera: Other insomnia treatment, suicidal ideation	Primary Outcome: ISI Secondary Outcome: CES-D, CI, PSWQ, REQ, REAQ, PSQI, sleep efficiency, days with insomnia, CSQ-8	2-arm RCT IC: Internet-based recovery training CC: Waitlist control condition	IC showed significantly greater improvement on primary outcome on posttest ($F(1,125) = 60.86$, $p < 0.001$; Cohen's d: 1.37 (0.99-1.77)) compared to CC. More participants in IC also showed symptom-free state compared to CC (OR: 6.78, CI = 1.87-24.62, $p < 0.001$). IC also showed significant effects for all secondary outcomes.	1b

**Gilbody (2017)[27]**	N = 369. Intervention: 187, control: 182, Male: 131, Female: 238, White British: 347, Other: 22. Inclusion criteria: PHQ-9 ≥ 10, English-speaking. Exclusion criteria: Active suicidality, diagnosis of psychotic depression, alcohol or drug abuse, recent delivery or bereavement	Primary Outcome: Depression diagnosis (PHQ-9 ≥ 10) Secondary outcome: PHQ-9, GAD-7, PHQ-15, EuroQol-EQ5D, use of resources (CSRI).	Multisite, open, two-arm, parallel-group RCT. Control Group: MoodGYM access. Intervention Group: MoodGYM + Telephone support	No longer having a PHQ-9 ≥10 was twice as high in the telephone-facilitated cCBT group compared to the control cCBT group (OR: 2.05, 95% CI: 1.23 to 3.42) at 4 months. This difference between groups was no longer significant at 12 months (OR 1.63, 95% CI 0.98 to 2.71, P = 0.06). Between-group differences followed a similar trend at 4-months (1.9, 95% CI 0.5 to 3.3, P = 0.009) compared to 12-months (0.9, 95% CI –0.5 to 2.3). No significant differences for GAD-7, PHQ-15, and EQ5D.	1b
**Gosling (2018)[28]**	N = 1149, SHUTi: 574, HealthWatch: 575, Male: 304,	Primary Outcome: GAD-7	Large-scale, two-arm RCT SHUTi: Access to	SHUTi had greater reduction in	1b

	Female: 844, Other; 1, No Ethnicity Data. Inclusion Critiera: BIS ≥ 3, subclinical depression (PHQ-9 > 4 and <20). Exclusion Criteria: shift workers, carers with sleep interruptions, pregnant women, no internet access, non-English speaking, diagnosis of psychosis, schizophrenia or bipolar, non-drug treatment for insomnia, suicidal ideation, medical reasons for insomnia	Secondary Outcome: DBAS-16, SAMI	SHUTi insomnia CBT website HealthWatch: Access to HealthWatch information website	GAD-7 than HealthWatch @ Post-test ($t_{724.27}$ = –6.77, CI: -2.44 to -1.34, $p < 0.001$) and at 6-month follow-up ($t_{700.67}$ = –4.27, CI: -1.79 to -0.66, $p < 0.001$). The effect was not moderated by dysfunctional beliefs about sleep.	
**Hagatun (2018)[29]**	N = 181, SHUTi: 95, Edu: 86, Male: 59, Female: 122, No ethnicity data. Inclusion Criteria: Age ≥ 18, diagnosis of insomnia. Exclusion Criteria: night work, other mental health/sleep condition	Primary Outcome: HADS, CFQ	Two-arm RCT SHUTi: Access to SHUTi insomnia CBT website Edu: Control group with access to a patient education website	High attrition rates (20% in SHUTi and 28% in Edu groups) Significantly larger decrease in HADS ($d_{between}$=0.57; 95% CI=0.79–0.35) and Fatigue ($d_{between}$=−0.92; 95% CI=1.22–0.62) in SHUTi group compared to Edu group (p	1b

				< 0.001 for both). Treatment gains were slightly reduced after 6-months.	
**Hagatun (2019)[30]**	N = 181. SHUTi: 95, Control: 86; Male: 59, Female: 122; No ethnicity data. Inclusion Criteria: Age ≥ 18, fluent in Norwegian, internet access, insomnia diagnosis (DSM-IV). Exclusion Criteria: night work, pregnancy, another sleep disturbance, mental health problem impairing sleep, other psychological treatment	Primary Outcome: ISI, BIS Secondary Outcome: Sleep diaries, DBAS-16, demographics, AUDIT-C, Internet Intervention Utility and Impact Questionnaires	Two-arm parallel RCT SHUTi: access to the SHUTi program Control: web-based patient education condition	SHUTi showed significant decrease in ISI (dbetween = –1.77, 95% CI = –2.23, –1.31) and BIS (dbetween = –1.00, 95% CI = –1.32, –.68) compared to control (p < 0.001 for both). Similar effect for DBAS-16 (p < 0.001; dbetween = –1.15, 95% CI = –1.44, –.86) and sleep diary measures (p = .13; dbetween = .17, 95% CI = –.04, .38). Adjusting for age, gender and years of education did not significantly alter results.	1b
**Lien (2019)[31]**	N = 178, CBTi: 92 (Morning: 41, Evening: 51), Edu: 86 (Morning: 44, Evening: 42),	Primary Outcome: MEQ Secondary Outcome:	Post-Hoc analysis of a two-arm parallel RCT (See Hagatun 2019 above). Both CBTi and Edu	Morning and Evening persons differed in favour of	1b

	Male: 59, Female: 119, No ethnicity data<br>Inclusion Criteria and Exclusion Criteria (See Hagatun 2019 above)	ISI, BIS, Sleep Diaries, DBAS-16, demographics, AUDIT-C, Internet Intervention Utility and Impact Questionnaires, HADS, CFQ	groups were divided between Morning and Evening persons.	morning on several MEQ measures (e.g. Wake after sleep onset (WASO) (d= 0.54, p < .001) and Early Morning Awakenings (EMA) (d= 0.38, p < .05)). Despite this, both groups in the CBTi condition improved comparably across secondary outcomes compared to Edu group (ISI Morning: $d_{between}$ =1.89 (CI = 1.12-2.48), p < .001; ISI Evening: $d_{between}$ =1.64 (CI = 1.01-2.15), p < .001 – similar results for BIA, DBAS, HADS, CFQ and sleep diary data).	
**Lintvedt (2013)[32]**	N = 163, Exp: 81, Control: 82, Male: 38, Female: 125, No ethnicity data<br>Inclusion Criteria: K10 ≥ 20, internet access	Primary Outcome: CES-D, ATQ, TDL, Secondary Outcome: User	Two-arm RCT<br>Exp: Internet intervention group<br>Control: Waitlist condition	Exp group showed lower levels of depressive symptoms, negative automatic	1b

		satisfaction		thoughts and improved depression literacy compared to control group (F[1,42] = 6.97, p<0.05, F[1,42] = 9.12, p<0.01 and F[1,41.28] = 5.61, p<0.05, respectively).	
**Lokman (2017)[33]**	N = 329, CDMI: 165, Control:164, Male: 80, Female: 249, No ethnicity data.<br>Inclusion Criteria: Age ≥ 18, internet access, speaks Dutch, adequate computer skills to participats, IDS-SR > 13 and < 39.<br>Exclusion Criteria: suicidal ideation by IDS-SR score	Primary Outcome: IDS-SR<br>Secondary Outcome: JSEQ, PSS, PSWQ, GAD-7, WEMWBS	Two-Arm RCT<br>CDMI: Complaint-directed mini-interventions group<br>Control: Waitlist group	Significant decrease in depression in CDMI group compared to control (mean –4.47, 95% CI –6.54 to –2.40; Cohen d=–0.70, P < 0.001), as well as in sleep problems, worry, anxiety and well-being (effect sizes between -0.29 to -0.40, P values between 0.007 and 0.03). Intervention did not reduce stress (–1.12 CI: –2.55 to 0.31, P = 0.12)	1b
**Loughnan (2019)[34]**	N = 87, iCBT = 43, TAU: 44, All Women, Australian: 63, Other: 14.	Primary Outcome: PHQ-9, GAD-7<br>Secondary	Two-arm RCT<br>iCBT: MUMentum CBT-based program<br>TAU: Treatment as usual, no other	iCBT produced moderate to large effect size reductions	1b

	Inclusion Criteria: Age ≥ 18, fluent in English, Australian, internet access, diagnosis of GAD or MDD, willing to provide info and GP's name, between 13 and 30 weeks pregnant. Exclusion Criteria: substance use or abuse, benzodiazepine use, schizophrenia or bipolar, current psychological therapy, suicidal ideation, severe depression	Outcome: K-10, EPDS, WHOQOL, MAAS, BDI-II, acceptability	treament	for anxiety on the GAD-7 (0.76, CI: 0.17 to 1.35, P < 0.05) and psychological distress K-10 (0.88, CI: 0.34 to 1.43, P < 0.05), superior to TAU group. No effect was seen for depression. Low adherence was noted (76%).	
**Mewton (2013)[35]**	N = 2413, Male: 859 Female: 1554, No ethnicity data. Inclusion Criteria: Diagnosis of MDD, GAD, panic disorder or social phobia Exclusion Criteria: suicidal ideation, drug or alcohol dependence, schizophrenia, bipolar, on atypical antipsychotics or benzodiazepines	Primary Outcome: K-10, WHODAS-II	Acceptability study. (Single group, all with access to iCBT program "This Way UP").	Older adults (60 years or above) were more likely to complete all six lessons than the younger groups. There were significant reductions in K10 (Beta: 7.6, SE: 0.5) and WHODAS-II (Beta: 4.3, SE: 0.5) post-intervention (p<0.001).	2c
**Moloney (2019)[36]**	N = 38, All Female, Caucasian: 36, Other: 2 Inclusion Criteria:	Primary Outcome: ISI, PSQI and interviews	Single-group, pre/post-test mixed-methods design	Positive, significant improvement (p < 0.01) in ISI (15.1 to	2b

	women, 45 and older, living in Appalachian Kentucky, difficulty sleeping (≥ 2 nights/week, for ≥ 3 months), current or previous use of sleep aids, internet access<br>Exclusion Criteria: self-reported obstructive sleep apnea, schizophrenia, dementia, Alzheimer’s, Cushing’s, or bipolar with psychosis	Secondary Outcome:<br>PSS, CESD-R-10. Medication use		6.5), PSQI (12.1 to 8.5), PSS (20 to 14.6) and CESD-R-10 (9.8 to 5.2). The odds of using medication was decreased as well (OR 0.28 [95% CI 0.11–0.74]).	
**Noguchi (2017)[37]**	N = 974, iCBT: 326, sEFM: 323, Waitlist: 325, Male: 486, Female: 488, No Ethnicity Data<br>Inclusion Criteria: CES-D ≥ 16 or PHQ-9 ≥ 5, Age > 19 and < 66, no suicidal ideation, internet access, have time to exercise 5-10 minutes twice per week for 5 weeks	Primary Outcome:<br>CES-D<br>Secondary Outcome:<br>PHQ-9, GAD-7	3-Arm RCT<br>iCBT: CBT-based online program group<br>sEFM: simple mindfulness exercise online group<br>Waitlist: waitlist control group	No significant differences between the intervention arms and the control group in references to CES-D (iCBT: -1.10, CI: -2.56, 0.37, p = 0.14; sEFM: -0.77, CI: -2.27, 0.73, p = 0.31). Significant difference in PHQ-9 in favor of sEFM group compared to control group (d = -1.12, CI: -1.98 to -0.27,	1b

				p = 0.01).	
**Proudfoot (2013)[38]**	N=720, Experimental: 231, Attention Control: 246. Waitlist: 228. Female: 491, Male: 229. No ethnicity data. Inclusion Criteria: Age 18-75, own a mobile phone and computer with internet access, 27-63 on DASS scale. Exclusion Criteria: answered positively to questions regarding suicide attempts/suicidal thoughts, or met criteria for psychosis based on PSQ questionnaire.	Primary Outcome: DASS21 Secondary Outcome: WSAS	3-Arm RCT Experimental: access to myCompass intervention modules Attention control: access to control mental health program. Waitlist: waitlist control group.	The intervention group showed significant improvement following the 7-week experimental period in comparison to both the waitlist (contrast estimate of 8.79, P=.000) and attention control group contrast estimate of 6.83, P=.000). Attention control and intervention group scores were not significantly different at the 3 month follow-up mark.	1b
**Romero-Sanchiz (2017)[39]**	N = 296.TSG[20] (I1): 98, LITG[21] (I2): 96, iTAU[22](C):102, Female: 224, Male: 72. No ethnicity data. Inclusion Criteria: Age 18-65, fluent in Spanish, 14-19 or 20-28 on BDI-II, symptoms lasting longer than 2 weeks, access to internet at home.	Primary Outcome: Cost utility and cost effectiveness , CSRI, cost of medication, OBLIKUE database (cost of medical test and services) Secondary Outcome:	Multicenter, 3-arm parallel RCT with economic evaluation. Control Group: iTAU, or care as usual Intervention 1: TSG, or CBT[25] alone, Intervention 2: LITG, or CBT + therapist support.	3 groups were comparable in cost during the year before baseline. Indirect costs were significantly higher in the LITG arm compared to other groups.TSG group's total net costs were	1b

	Exclusion Criteria: Psychological treatment (past year), severe Axis I psychiatric disorder, increased AD meds.	BDI-II (Spanish version), EQ5D		lower (€700-800) than the other two groups (higher indirect costs in LITG, higher direct costs in iTAU),1-point improvement in BDI-II using TSG instead of iTAU was accompanied by savings of €169.50 LITG compared to iTAU showed savings of €104.63. Each QALY[27] for TSG instead of iTAU also saved €11389.96, while LITG to iTAU saved €6380.96.	
**Van Kessel (2016)[40]**	<u>N = 39</u>, Plus: 19, Control: 20, Male: 10 Female: 29, European: 39, no others. Inclusion Criteria: Diagnosis of multiple sclerosis, still ambulatory for at least 100m, CFQ ≥ 4, abstain for new treatment, New Zealand resident	<u>Primary Outcome:</u> CFQ, MFIS <u>Secondary Outcome:</u> HADS	Two-arm RCT Plus: Access to MSInvigor8 and regular email support from a therapist Control: Access to MSInvigor8 only	Plus group showed significantly greater reductions in fatigue ($F_{[1,36]} = 9.09$, $p < 0.01$) compared with Control group. No significant differences in anxiety and depression between groups.	1b

ACQ: Agoraphobic Cognitions Questionnaire; AD: Antidepressant; ASI: Anxiety Sensitivity Scale; ATQ: Automatic Thoughts Questionnaire; AUDIT-C: Alcohol Use Disorders Identification Test Consumption; BAI: Beck Anxiety Inventory; BDI-II: Beck Depression Inventory II; BIS: Bergen Insomnia Scale; BPD: Bipolar Disorder; BSI: Brief Symptom Inventory; BSQ: Body Sensations Questionnaire; cCBT: Computerized Cognitive Behavioral Therapy; CDMI: Compliant-Directed Mini—Interventions; CES-D: Centre for Epidemiologic Studies-Depression scale; CESD-R-10: Center for Epidemiologic Studies Depression Scale-Revised; CFQ: Chalder Fatigue Scale; CI: Cognition Irritation Scale; CSQ-8: Client Satisfaction Questionnaire ; CSRI: Client Service Receipt Inventory; CVD: Cardiovascular Disease; DASS-21: Depression Anxiety Stress Scales – Short Form; DBAS-16: Dysfunctional Beliefs About Sleep-16 Scale; DSM-IV: Diagnostic and Statistical Manual of Mental Disorders, fourth edition; EPDS: Edinburgh Postnatal Depression Scale; EuroQol-EQ5D: European Health-Related Quality of Life; GAD: Generalized Anxiety Disorder; GAD-7: Generalized Anxiety Disorder 7-Item Scale; HADS: Hospital Anxiety and Depression Scale; IDS-SR: Inventory of Depressive Symptomatology Self-Report; IIP: Interpersonal Problems; ISI: Insomnia Severity Index; iTAU: Improved Treatment as Usual; JSEQ: Jenkins Sleep Evaluation Questionnaire; K-10: Kessler Psychological Distress Scale; LITG: Low-intensity Therapist Guided; LSAS: Liebowitz Social Anxiety Scale; MAAS: Maternal Antenatal Attachment Scale; MDD: Major Depressive Disorder; MEQ: Horne-Östberg Morningness Eveningness Questionnaire; MFIS: Modified Fatigue Impact Scale; MIA: Mobility Inventory for Agoraphobia, Avoidance Alone; MIB: Mobility Inventory for Agoraphobia, Avoidance Accompanied; MINI: Mini international Neuropsychiatric interview; OASIS: Overall anxiety severity and impairment scale; PANAS: Positive and Negative affect scale; PDA: Panic Disorder; PHQ-9: Patient Health Questionnaire 9-item scale (for depression); PHQ-15: Patient Health Questionnaire 15-item scale (for somatoform disorders); PSQI: Pittsburgh Sleep Quality Index; PSS: Perceived stress scale; PSWQ: Penn State Worry Questionnaire; QALY: Quality Adjusted Life Year; RCT: Randomized Controlled Trial; REAQ: Recreation Experience and Activity Questionnaire; REQ: Recovery Experiences Questionnaire; SAD: Social Anxiety Disorder; SAMI: Sleep-Associated Monitoring Index; SCID: Structured Clinical Interview for DSM-IV; SD: Standard Deviation; SF-12: Short-Form Health Survey-12; SIAS: Social Interaction Anxiety Scale; SPS: Social Phobia Scale; SUS: System Usability Scale; TAU: Treatment as Usual; TDL: Treatment depression literacy; TSG: Totally Self-Guided; WEMWBS: Warwick-Edinburgh Mental Well-being Scale; WHODAS-II: World Health Organisation Disability Assessment Schedule; WHOQOL: World Health Organization Quality of Life; WHOQOL-BREF: World Health Organization Quality of Life (Abbreviated)
