# Supplementary material for: Self-directed Technology-Based Therapeutic Methods for Adult Patients Receiving Mental Health Services: Systematic Review
Source: JMIR Ment Health. 2021 Nov 26;8(11):e27404. doi: 10.2196/27404 (PMC8665378; doi:10.2196/27404)
Supplement: Multimedia Appendix 2 [file mental_v8i11e27404_app2.pdf]

Table 4. Summary of the data extracted from non-ICBT studies.

Randomized Controlled Trials					
**Author (Year)**	Sample	Outcomes	Evaluation	Results	LOE
**Aardoom (2016)[41]**	N=354, Intervention: 87, I + low therapist support: 88, I + high therapist support: 89. Control: 90, Male: 4, Female: 350, no ethnicity data. Inclusion Criteria: Age ≥ 16, access to internet, and eating disorder (ED) symptoms	Primary outcome: SEED, EDE-Q Secondary outcome: ED-QOL, PHQ-4 and PTQ, User-satisfaction questionnaire (4-point Likert Scales).	4-arm RCT. Groups:1-Featback, 2-Featback with low-intensity therapist support (1x/week, 20-minute session), 3-Featback with high-intensity therapist support (3x/week, 20-minute sessions), 4-Control (Waiting List)	Intervention vs. waiting list: Time-by-condition effects were found for ED psycho-pathology (d=-0.16, 95% CI = -0.31 to -0.01), depression (d=−0.31, 95% CI=−0.54 to −0.09), and perseverative thinking (d=−0.28, 95% CI=−0.45 to −0.11).No significant differences reported between interventions. Higher satisfaction in therapist support groups.	1b
**Bernstein (2016)[42]**	N = 60, Intervention: 30, Control:30, Male: 30 Female: 30, Caucasian: 27, African American: 27, Hispanic:13 other: 6. Inclusion Criteria: Age ≥ 18, English-speaking, cell phone with texting, tobacco use, lifetime use ≥ 100 cigarettes.	Primary Outcome: Self-reported 7-day abstinence of tobacco at 1 and 3 months. Secondary Outcome: Associations between EMA responses and tobacco abstinence, adherence to EMA and abstinence.	Prospective, two-arm RCT. Intervention group received brochure, nicotine replacement therapy (NRT), Quitline Referral, and SmokefreeTXT messages, Control Group: only received brochure.	One-month tobacco abstinence: 47% (14) of intervention group vs. 10% (3) in control group, three-month tobacco abstinence:30% (9) of intervention group vs. 13% (4) in control group, one month: Responding to texts increased likelihood of being abstinent (3.8 texts in abstinent vs. 0.4 texts in continued smoking group). Not significant at 3	2b

				months (3.6 texts vs. 1.3 texts)	
**Constant (2014)[43]**	N = 469. Intervention: 234, Control: 235, women only, No ethnicity data. Inclusion criteria: Women to undergo medical abortion, age ≥ 18, access to phone with texting	Primary Outcome: HADS, Adler's 12-item emotional scale for abortion settings. Secondary Outcome: IES-R (subjective stress).	Multisite, 2-arm RCT. Outcome assessment included acceptability of intervention (blinding not possible)	Anxiety by HADS was lower in the intervention group (β=1.3; 95% CI=0.3 to 2.4; p=0.013). 98% of the intervention group said that the messages helped them through the procedure. 99% stated that they would recommend the messages to a friend.	1b
**Kannisto (2017)[44]**	N = 1139. Intervention: 563, Control: 560, Male: 560, Female: 579. Finnish study: no ethnicity data. Inclusion Criteria: Age ≥ 16, prescription for antipsychotic medication, access to mobile phone, Finnish-speaking. Exclusion Criteria: Forensic patients, patients with a planned non-acute treatment period	Primary Outcome: Recruitment measured as those who were screened, eligible, consent not asked, refused, and enrolled. Attrition - measured by dropout rate during message intervention, 12-month, participant notifications, postal surveys and register data. Secondary Outcome: Quality of Life and satisfaction	Multicenter, 2-arm RCT with randomization. Intervention group - message intervention + usual treatment Control Group: usual treatment only	11,530 were assessed.4186 (36.31%) were eligible based on criteria. 3417 were asked consent. 2278 (66.67%) refused, with 1139 remaining. 16 were lost before start. Of the 563 in the intervention group, 27 (4.8%) dropped-out during the 12-month intervention. Of the 558 contacted by phone at 12-months, 155 (27.8%) did not respond. 35 participants (6.2%) withdrew from follow-up.589 (52.45%) did not respond to postal survey.	1b

		(Q-LES-Q and CSQ-8)			
**Kleiboer (2015)[45]**	N = 537, C1:107, C2: 108, C3: 106, C4: 110, C5: 106, Male: 187, Female: 348, No ethnicity data. Inclusion Criteria: Age ≥ 18, CES-D > 15 and < 40, HADS > 7 and < 16. Exclusion Criteria: insufficient knowledge of Dutch language, suicidal ideation, mental health treatment by a specialist	Primary Outcome: CES-D, HADS Secondary Outcome: BAI, PHQ-9, CSQ-8	5-arm RCT C1: PST therapy without support, C2: PST therapy with support on request, C3: PST therapy with weekly support, C4: No treatment, non-specific support, C5: access to psycho-education website about depression/anxiety	Small but significant between-group improvement in depressive (CESD: 0.34, (0.07-0.61), $p < 0.01$) and anxiety (HADS: 0.30 (0.02-0.57), $p < 0.05$) symptoms favoring the C3 group (weekly support). BAI showed no significant between group differences. All groups showed improvement post-treatment for all measures ($p < 0.05$).	1b
**Mason (2012)[46]**	N = 1758. Intervention: 877, Control: 881, Female: 1126, Male: 632, White: 1642, Black:21 Asian: 62, Other: 33. Inclusion Criteria: Age ≥18, based in the UK, cigarette or roll-up smokers.	Primary Outcome: Self-reported 3 months prolonged abstinence. Secondary Outcome: one month prolonged abstinence, 7-day and 24-hour point prevalence abstinence, Questionnaire (email or phone) on recall, readability, usefulness, other sources of support	2-arm RCT Intervention group: Tailored advice report + tailored progress report Control Group: Only the standard report	No differences on self-reported abstinence at 6-month follow-up or secondary outcome measures related to abstinence.Of those abstinent, 59.4% said they quit as a result of the report, with more from the intervention group (67.1% versus 49.1%). Intervention participants rated higher scores on ‘easy to read and understand’, and ‘was written for me’. No difference between groups on seeking extra	1b

		used.		services.	
**Pictet (2016)[47]**	N = 101, ICBM: 34, CCBM: 34, Waitlist: 33, Male: 21, Female: 80, No ethnicity data. Inclusion Criteria: BDI-II ≥ 14, Age 18 to 65, fluent in French, internet access. Exclusion Criteria: Current psychological therapy or change in antidepressant med < 1 month	Primary Outcome: BDI-II Secondary Outcome: SUIS, Anhedonic scale based on BDI-II, STAI-T, AST-D-II, SHAPS, TEPS	3-arm RCT ICBM: Imagery-based cognitive bias modification group CCBM: CBM group with imagery component Waitlist: no intervention provided during the study	ICBM showed greater improvements in depressive symptoms than CCBM group (d = 0.86, 95% CI = [0.33, 1.3]) and Waitlist group (d = 1.17, 95% CI = [0.62, 1.65]), with no significant difference between CCBM and Waitlist. ICBM and CCBM showed improvements in Anhedonia; Waitlist did not, sometimes showing a decrease (i.e. TEPS). (P-values not reported for between group differences)	1b
**Sherman (2012)[48]**	N = 249. Approx. 60 per group (4). All Female. White: 148, African American: 33, Hispanic: 19, Asian/Pacific Islander: 11, Other: 3. Inclusion Criteria: Female, early-stage breast cancer, enrolled in one of four oncology services in study, no prior history of cancer,	Primary Outcome: PAL-C, SRHS, PAIS , Breast Cancer Treatment Response Inventory. Secondary Outcome: Side effect incidence, primary medical data, demographics.	4-arm, multicenter review of RCT data .Group 1 (Control): usual care. Group 2: usual care + psycho-educational videos. Group 3: telephone counseling . Group 4: videos + telephone counseling	All groups showed improvement over time in overall health, psychological well-being and social adjustment. No significant differences in physical adjustment. Poorer emotional adjustment over time in control group compared to 3 intervention groups, marked by increased side effect distress in the control group (p=0.012).	2b

	"partner", English-speaking, no uncontrolled chronic condition or history of psychiatric hospitalization or drug abuse.			Difference between 3 groups was not statistically significant. Group 3 showed marked decline in psychological well-being from adjuvant phase through ongoing recovery phase. No significant differences in social adjustment.	
			**Non-RCT Quantitative Studies**		
**Ahmedani (2015)[49]**	N= 75. Completed program: 69, Follow-up w/ phone call: 64. Male: 21, Female: 43 Caucasian: 34, African American: 19, Other groups: 11. Inclusion criteria: PHQ-9 ≥ 5, age ≥ 18, complaint of chronic pain.	Primary Outcome: PHQ-9 Secondary Outcome: ODI, FABQ-PA, satisfaction questionnaire	Pilot feasibility study with pre-posttest design.	Mean PHQ-9 at baseline: 12.4. Two-week follow-up: 10.2 8.1% reduction (95 CI: -3.4 to -0.9, p= 0.001). Post-intervention, 35.3% of moderate depression and 31.8% of mild depression had decreased to the lower categories by two weeks. No reported CI's on ODI and FABQ-PA. 76.6% liked the tablets, 85.9% reported the patient videos highly, 60.9% reported interest in addressing their depression as a result of the program.	2b
**Kipping (2016)[50]**	N = 3158. Portal Users: 461. Male: 1402, Female: 1756. No ethnicity data. Inclusion Criteria: All patients receiving care from December 2014 to December 2015 at Ontario Shores and proxy users.	Primary outcome: Benefits evaluation - portal usage, appointment keeping, etc. Secondary Outcome: MHRM, System and Use surveys - e-visits (with clinician), e-views (health records, appointments), e-requests (script refills), surveys	Observational cohort study. Randomization and blinding were not used.	Portal accessed 4761 times. E-views: n=4359, 95.3% E-visits: n=210, 4.4%; E-renewals: n=12, 0.3% 2014: odds of user attending scheduled appointment were 17% greater than nonusers (OR 1.17, 95% CI 1.08-1.26). 2015: odds of user attending scheduled appointment were 67% greater than nonusers (OR 1.67, 95% CI 1.56-1.79). MHRM increased from 70.4 to 81.7 at follow-up (p=0.1) in users. Qualitative comments: psychologists not uploading their reports, no history of visits, easy to use, good tool to cultivate autonomy, helpful for reminders, saves time/money, satisfaction with system.	2b
**Piette (2013)[51]**	N = 1173, Patients with Depression: N = 442. Male:	Primary Outcome: IVR Call	4-arm Retrospective Cohort study	Depression had the lowest call completion rates (CHF:90%, cancer: 90%, diabetes: 81% and depression:	2b

	110, Female: 332. White: 399, Other: 43. Inclusion Criteria: English-speaking, diagnosis of depression. Exclusion Criteria: Cognitive impairment, (e.g. Schizophrenia), limited life expectancy, majority of care not at recruitment site, unable to respond to IVR[5]	Completion Secondary Outcome: Demographics		71%, p<0.001). Call completion rates decreased over time among patients in the depression program, despite increasing in other groups (i.e. heart failure) Lower mental health summary scores at enrollment, one or more missed clinical appointment, prior hospitalization, and increasing weeks of program participation were associated with lower odds of completing a weekly call.	
**Pratap (2018)[52]**	***N = 345 (274), EVO: 83, iPST: 112, HTips: 79, Male: 79, Female: 266, Hispanic: 106, Non-Hispanic White: 184, African-American: 25, American Indian/Alaskan Native: 3, Asian: 24, Other: 3 Inclusion Criteria:English or Spanish speaking, Age ≥ 18, iPhone or Android device with iPad (compatible with program), PHQ-9 ≥ 5 or PHQ-9 item 10 ≥ 2 (feeling disabled).	Primary Outcome: PHQ-9, SDS Secondary Outcome: Demographic characteristics, engagement statistics	Three-arm Feasibility with partial randomization (participants chose two favorites, than randomized) EVO: cognitive-based control application iPST: internet-based problem solving application HTips: information control over depressed mood through self-care and physical activity	Sample of 345 dropped to 274 over the course of the study, with a larger and quicker dropout among Hispanic/Latino participants. No significant differences were noted between different ethnicities in regards to treatment outcomes. While depressive symptoms and functional impairment improved for all three treatment arms ((beta=–2.66 (SE = 0.96, P=.006), outcomes did not vary significantly by treatment.	2b
**Stein (2012)[53]**	N = 1122. Exposed: 122, Unexposed: 1000. Male: 397, Female: 725. Caucasian: 855, Minority: 267. Inclusion Criteria: Medicaid-only eligible adults who received an antipsychotic, antidepressant or mood-stabilizing psychotropic medication.	Primary Outcome: 180-day medication adherence. Secondary Outcome: Demographics	Case Control Study Unexposed: Participants who did not use CommonGround who had received psychotropic medication. Exposed: those that had used the decision support center and CommonGround two or more	After controlling for demographic and clinical variables, it was determined that using CommonGround did not influence medication adherence rates. There was a slight increase in adherence among users, but it was less than the increase among non-users.	3b

times.					

Qualitative Study

Author (Year)	Sample	Outcomes	Evaluation	Results	LOE*
**Bauer (2018)[54]**	N = 17. Male: 7, Female: 10. White: 16, African American: 1. Inclusion Criteria: English-speaking, adults diagnosed with depression or anxiety Exclusion criteria: Active suicidality, current diagnosis of dementia, substance dependence, bipolar disorder, psychotic disorder	Primary Outcome: Participant usage Secondary Outcome: PHQ-9[1] and GAD-7[2] surveys, Acceptability and satisfaction scores by questionnaires.	Pilot feasibility study. Randomization not suitable No blinding conducted.	All participants used the app for the first 4 weeks. Only 6 used it through 8 weeks. Weekly PHQ-9 and GAD-7 scores response rates ranged from 86 to 100%. 88% of participants completed all measures. Response rate for medication surveys ranged from 30 to 67%. 100% said the app was easy to use. 77% reported satisfaction with the app and 67% said it was useful. 46% reported feeling more connected with their doctor. 13% reported negative views on the app and 56% were neutral on privacy issues.	5

AST-D- II: Ambiguous Scenarios Test for depression-related bias-II; BAI: Beck Anxiety Inventory; CES-D: Centre for Epidemiologic Studies-Depression Scale; CHF: Congestive Heart Failure; CSQ-8: Client Satisfaction Questionnaire; ED-QOL: Eating-Disorder related Quality of Life; EDE-Q: Eating Disorder Examination Questionnaire; EMA: Ecological Momentary Assessment; FABQ-PA: Fear and Avoidance Beliefs Questionnaire – Physical Activities subscale; GAD-7: Generalized Anxiety Disorder 7-item Scale; HADS: Hospital Anxiety and Depression Scale; IES-R: Impact of Event Scale-Revised; IVR: Integrated Voice Response; LOE = Level of Evidence (according to the Oxford Centre for Evidence-based Medicine Levels of Evidence[18]); ODI: Oswestry Disability Index; MHRM: Mental Health Recovery Measure; PAIS: Psychosocial Adjustment to Illness Scale; PAL-C: Profile of Adaptation to Life Clinical Scale; PHQ-4: 4-item Patient Health Questionnaire; PHQ-9: Patient Health Questionnaire 9-item Scale (for depression); PTQ: Perseverative Thinking Questionnaire; SDS: Sheehan Disability Scale; SEED: Short Evaluation of Eating Disorders; SHAPS: Snaith-Hamilton Pleasure Scale; SRHS: Self-Report Health Scale; STAI-T: Trait Scale of State-Trait Anxiety Inventory; SUIS: Spontaneous Use of Imagery Scale; TEPS: Temporal Experience of Pleasure Scale
